# Characterization of Amide Bond Conformers for a Novel Heterocyclic Template of *N*-acylhydrazone Derivatives

**DOI:** 10.3390/molecules181011683

**Published:** 2013-09-25

**Authors:** Alexandra Basilio Lopes, Eduardo Miguez, Arthur Eugen Kümmerle, Victor Marcos Rumjanek, Carlos Alberto Manssour Fraga, Eliezer J. Barreiro

**Affiliations:** 1 Laboratório de Avaliação e Síntese de Substâncias Bioativas (LASSBio), Universidade Federal do Rio de Janeiro, CCS, Cidade Universitária, P.O. Box 68.023, Rio de Janeiro 21941-902, RJ, Brazil; E-Mail: alexandralopesb@yahoo.com.br; 2 Programa de Pós-Graduação em Química, Instituto de Química, Universidade Federal do Rio de Janeiro, Rio de Janeiro 21949-900, RJ, Brazil; 3 Instituto de Macromoléculas Professora Eloisa Mano, Universidade Federal do Rio de Janeiro, Cidade Universitária, Ilha do Fundão, Rio de Janeiro CEP 21941-598, RJ, Brazil; E-Mail: emiguez@ima.ufrj.br; 4 Departamento de Química, Instituto de Ciências Exatas, Universidade Federal Rural do Rio de Janeiro, Seropédica 23.890-000, RJ, Brazil; E-Mails: akummerle@hotmail.com (A.E.K.); victor.rumjanek@gmail.com (V.M.R.); 5 Programa de Pesquisa em Desenvolvimento de Fármacos, Instituto de Ciências Biomédicas, Universidade Federal do Rio de Janeiro, PO Box 68023, Rio de Janeiro 21941-902, RJ, Brazil

**Keywords:** *N*-acylhydrazones, pyrimidines, conformers, stereochemistry, NMR

## Abstract

Herein we describe NMR experiments and structural modifications of 4-methyl-2-phenylpyrimidine-N-acylhydrazone compounds (aryl-NAH) in order to discover if duplication of some signals in their ^1^H- and ^13^C-NMR spectra was related to a mixture of imine double bond stereoisomers (*E*/*Z*) or CO-NH bond conformers (*syn* and *anti-periplanar*). NMR data from NOEdiff, 2D-NOESY and ^1^H-NMR spectra at different temperatures, and also the synthesis of isopropylidene hydrazone revealed the nature of duplicated signals of a 4-methyl-2-phenylpyrimidine-N-acylhydrazone derivative as a mixture of two conformers in solution. Further we investigated the stereoelectronic influence of substituents at the *ortho* position on the pyrimidine ring with respect to the carbonyl group, as well as the electronic effects of pyrimidine by changing it to phenyl. The conformer equilibrium was attributed to the decoplanarization of the aromatic ring and carbonyl group (generated by an *ortho-*alkyl group) and/or the electron withdrawing character of the pyrimidine ring. Both effects increased the rotational barrier of the C-N amide bond, as verified by the Δ*G*^≠^ values calculated from dynamic NMR. As far as we know, it is the first description of aryl-NAH compounds presenting two CO-NH bond- related conformations.

## 1. Introduction

**Scheme 1:**
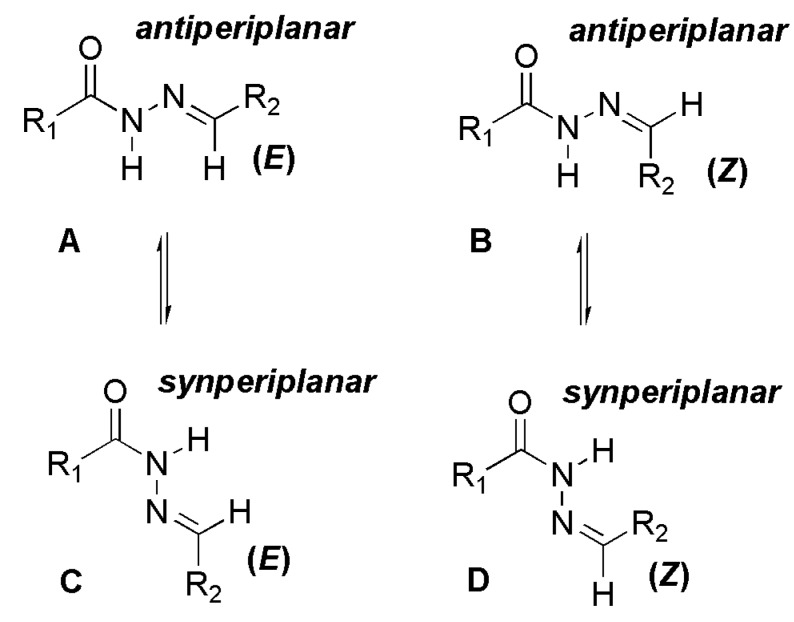
General structure and stereochemistry of NAH.

The bioactive *N*-acylhydrazone (NAH) moiety has been identified in a great number of lead compounds that act on different types of molecular targets [1,2,3,4]. Because of the assemblage of amide and imine functions, NAH compounds may exist as C=N double bond stereoisomers (*E*/*Z*) (Scheme 1) and as *syn/antiperiplanar* conformers about the amide CO-NH bond (Scheme 1) [5].

**Figure 1:**
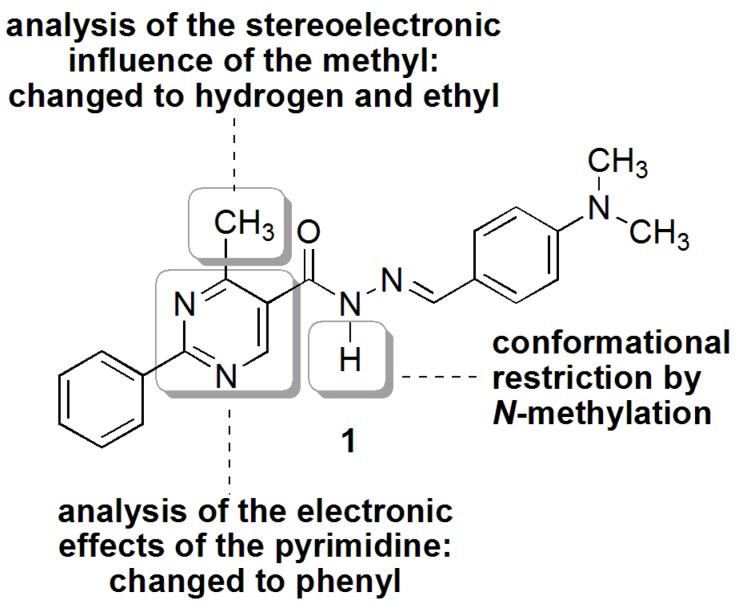
Stereoelectronic modifications of the NAH derivative LASSBio-1083 (**1**).

A research program to develop a series of 2-phenyl-4-methylpyrimidine-*N*-acylhydrazone compounds with antinociceptive and anti-inflammatory activities led to the discovery of LASSBio-1083 (**1**, Figure 1), a promising lead compound that shows an ED_50_ of 27 µmol/Kg in the acetic acid-induced mouse writhing test [6]. Regarding full structural characterization, the ^1^H-NMR spectra revealed the duplication of some signals, indicating the presence of a mixture of stereoisomers or conformers. 

Using ^1^H- and ^13^C-NMR data, Palla and coworkers posited that NAH compounds derived from the condensation of hydrazides and aromatic aldehydes are present in solution as the *E* geometric isomer, which is less sterically hindered compared to the *Z* form [7]. However, starting from the pyridine-2-carboxaldehyde, the *Z* isomer can be detected in less polar solvents due to its stabilization with intramolecular H-bonds [5]. On the other hand, the duplication of NMR signals has been attributed to the presence of *anti-* and *synperiplanar* conformers in benzyl and alkyl NAHs [8,9,10], but there is no description of this phenomenon in aryl NAHs in the literature. Because the complete knowledge of structure, including stereochemistry, is essential for lead optimization in drug discovery, we herein describe our studies concerning the elucidation of the signal duplication in the ^1^H-NMR of **1** and the modifications of its structure leading to compounds **2a**–**d** and **3a** to better understand the stereoelectronic properties that promote this effect (Figure 1).

## 2. Results and Discussion

### 2.1. Chemical Synthesis

#### 2.1.1. Synthesis of Esters **4a**–**e**

**Scheme 2:**
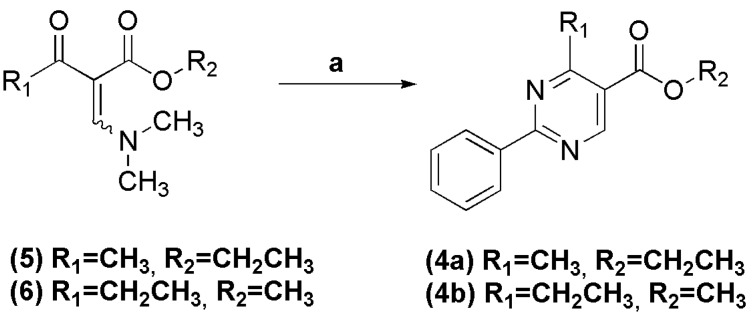
Synthesis of 4-alkyl-2-phenylpyrimidinyl esters **4a** and **4b**.

Esters **4a** and **4b** were chemoselectively obtained in 80% and 73% yield, respectively, through the condensation of benzamidine with (*Z*/*E*)-ethyl 2-((dimethylamino)methylene)-3-oxobutanoate (**5**) and (*Z*/*E*)-methyl 2-((dimethylamino)methylene)-3-oxopentanoate (**6**), respectively, in ethanol (for **4a**) and methanol (for **4b**) at room temperature (Scheme 2) [11,12]. The synthesis of the pyrimidine nucleus was confirmed by the presence of singlets at 9.17 and 9.12 ppm in the ^1^H-NMR spectra, which refer to the H-6 attached to the pyrimidine ring in esters **4a** and **4b**, respectively.

*Reagents and conditions*: (a) for **4a**: benzamidine hydrochloride, Na/EtOH, rt, 2 h, 80% and **4b**: benzamidine hydrochloride, NaOH/MeOH, rt, 1 h, 73%.

**Scheme 3:**
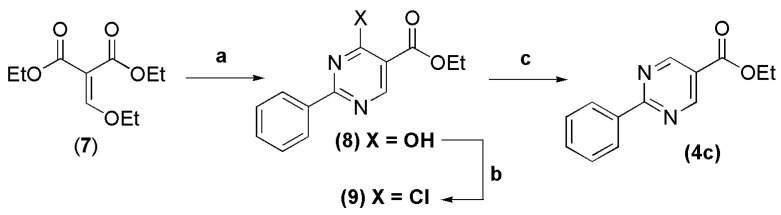
Synthesis of unsubstituted 2-phenylpyrimidinyl ester **4c**.

Ethyl 4-hydroxy-2-phenylpyrimidine-5-carboxylate (**8**) was obtained in 50% yield by condensation between benzamidine and diethyl 2-(ethoxymethylene)malonate (**7**) in ethanol at room temperature (Scheme 3) [12]. Two subsequent reactions from **8** furnished the ethyl ester **4c** in 50% overall yield. The first step involved chlorination of **8** using POCl_3_ at 100 °C, and the second one was dehalogenation of **9** by a radical reaction using zinc and AcOH in THF (Scheme 3) [13,14]. The presence of a singlet at 9.22 ppm that integrated to 2H, confirmed the dehalogenation of C-4 and formation of the pyrimidinyl ester **4c**.

*Reagents and conditions*: (a) benzamidine hydrochloride, Na/EtOH, rt, 9 h, 50%; (b) POCl_3_, 100 °C, 1 h, 98%; (c) Zn, AcOH, THF, 60 °C, 23 h, 50%.

**Scheme 4:**
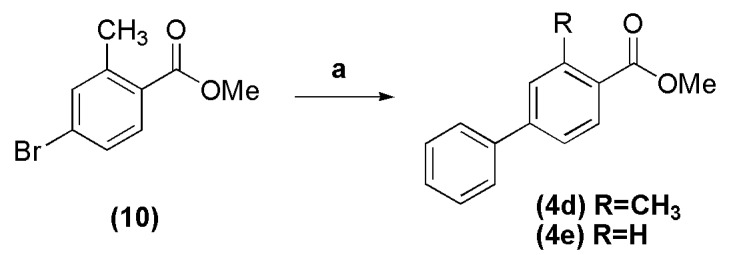
Synthesis of biphenyl esters **4d** and **4e**.

The synthesis of biphenyl ester **4d** was achieved by Suzuki cross-coupling between methyl ester **10** and phenylboronic acid in the presence of potassium carbonate and PdCl_2_(PPh_3_)_2_ at 80 °C in 91% yield, while the ester **4e** was obtained commercially (Scheme 4) [15].

*Reagents and conditions*: (a) phenylboronic acid, PdCl_2_(PPh_3_)_2_, K_2_CO_3_, toluene/MeOH, 60 °C, 3 h, 91%.

#### 2.1.2. Synthesis of *N*-acylhydrazone Derivatives **1**–**3**

**Scheme 5:**
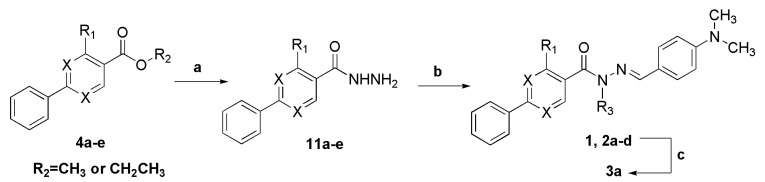
Synthesis of functionalized *N*-acylhydrazone derivatives **1**, **2a**–**d** and **3a**.

The key precursors for the synthesis of NAH **1** and **2a**–**d**, the phenylpyrimidine and biphenyl hydrazides **11a**–**e**, were obtained in 79% to 95% yield by nucleophilic acyl substitution reaction of **4** with 100% hydrazine hydrate in ethanol at 55–80 °C (Scheme 5) [16]. The desired compounds **1** and **2a**–**d** were synthesized using the classic acid-catalyzed condensation of hydrazides **11a**–**d** with 4-dimethylaminobenzaldehyde in ethanol at room temperature in 85%–99% yield (Scheme 5 and Table 1). Analysis of the ^1^H-NMR in DMSO-*d*_6_ showed the formation of NAH **1** and **2a**–**d** by the presence of signals attributed to the N=CH and CONH protons, but for some NAH (**1**, **2a**–**c**), these signals appear to be duplicated (Table 1). Finally, the *N*-methyl-NAH derivatives **3a** were obtained from a selective *N*-methylation of **1** using methyl iodide in a basic medium of K_2_CO_3_ at 43 °C in 77% yield (Scheme 5 and Table 1) [17]. The presence of signals referring only to the N-CH_3_ protons indicates the selective *N*-methylation of NAH. Additionally, no signal duplication was observed for these *N*-methyl-NAH derivatives.

*Reagents and conditions*: (a) NH_2_NH_2_.H_2_O, 55–80 °C, 2–8 h, 79%–95%; (b) 4-(dimethylamino)benzaldehyde, catalytic HCl, EtOH, rt, 85%–99%; (c) CH_3_I, K_2_CO_3_, acetone, 43 °C, 24 h, 77%.

**Table 1:** Synthesis of *N*-acylhydrazone and *N*-methyl-*N*-acylhydrazone derivatives **1**–**3**.

Compound	X	R_1_	R_3_	Yield (%) ^a,b^	Ratio a:b
**1**	N	CH_3_	H	66	2:1
**2a**	N	H	H	21	4:1
**2b**	N	CH_2_CH_3_	H	49	1.7:1
**2c**	C	CH_3_	H	81	5:1
**2d**	C	H	H	84	^c^
**3a**	N	CH_3_	CH_3_	51	^c^

^a^ Global yield; ^b^ The analytical results for C, H and N were within 0.4% of the calculated values; ^b^ Ratio obtained by signal integration of the duplicate N=CH peaks in the ^1^H-NMR spectra; ^c^ Only one signal is present.

### 2.2. NAH Stereochemistry Elucidation

#### 2.2.1. Determination of the Relative Configuration of the Imine Double Bond

**Figure 2:**
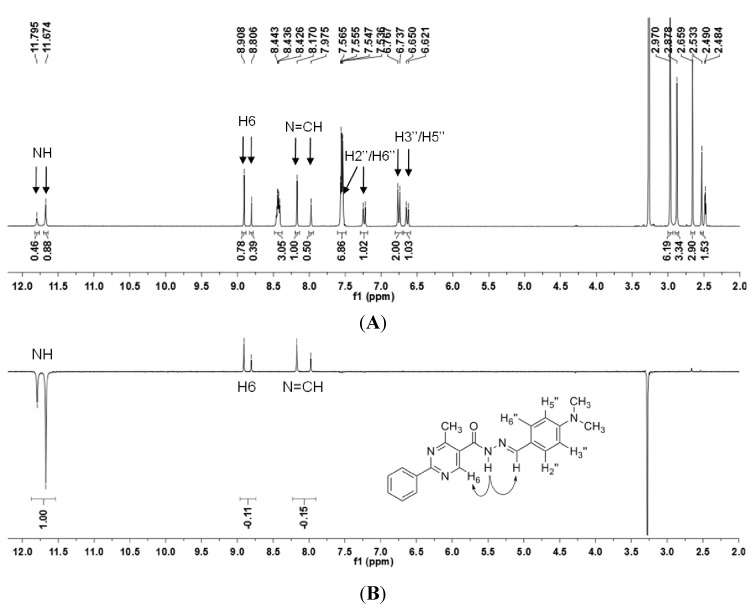
(**A**) ^1^H-NMR and (**B**) NOEdiff spectra for irradiation of NH (the major peak) of NAH derivative **1**.

The ^1^H-NMR spectrum of the analgesic lead compound LASSBio-1083 (**1**) is presented in Figure 2A and shows the duplication of the hydrogen signals. Assuming that these duplications could be attributed to the presence of the two possible isomers (*E*/*Z*) of the imine double bond, we decided to proceed with differential Nuclear Overhauser Effect (differential NOE) experiments to assess the spatial proximity of ^1^H-^1^H. The hydrogen atom selected for irradiation was the NH of the amide. Due to the presence of two singlets related to the NH hydrogen, we chose the one that shifted less and that was more prevalent at 11.67 ppm (Figure 2B).

Although only one amide signal at 11.67 ppm was selected for irradiation in the NOE experiments, the amide hydrogen at 11.79 ppm presented the same irradiated signal phase. Furthermore, both signals for H_6_ and N=CH showed increased intensities, presenting a positive NOE of 11% and 15%, respectively (Figure 2B).

Because of the angle and distance, a positive NOE on N=CH was not expected from NH irradiation of the *Z* isomer. Calculations using Mspin 1.03 software [18] were performed to simulate the NOE effect at a radius of 5 Å around the irradiated hydrogen. For this simulation, more stable *E* (**A**/**B**) and *Z* (**C**) stereoisomers, obtained through a process of geometric optimization by molecular mechanics followed by conformational analysis using the semi-empirical method AM1 in the PC Spartan Pro 1.0.5 software [19], were selected (Table 2). Although the **B** conformer was not as stable as **A**, the choice was based on observations from other NAH compounds in X-ray diffraction studies (Table 2) [17].

The theoretical results for amide NH irradiation showed that this effect should be observed mainly in H_6_ and N=CH for both *E* (**A** and **B**) conformers and be more pronounced at N=CH in the **B** conformer (Table 2). The same analysis for the *Z* stereoisomer showed that the greatest positive NOE should be observed at H_2_” followed by H_6_, but no significant positive NOE would be expected for N=CH (Table 2).

**Figure 3:**
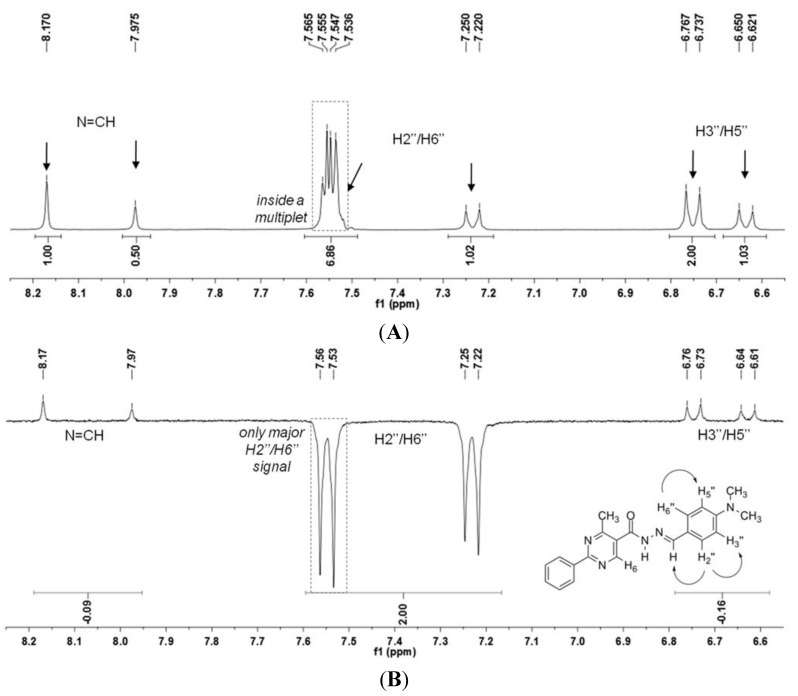
(**A**) Expanded ^1^H-NMR and (**B**) NOEdiff spectra for irradiation of H_2_”/H_6_” (in minor proportion) of NAH derivative **1**.

To clarify the reason for the negative phase of the minor NH signal and exclude the possibility of a technical artifact due to the proximity of signals, we decided to irradiate the signal of the less prevalent H_2_”/H_6_” at 7.20 ppm. This location was relatively distant from the multiplet and doublet neighbor signals, whereas the signal corresponding to H_2_′′/H_6_′′ in greater proportion was located inside a multiplet at 7.54 to 7.57 ppm (Figure 3). The irradiation led again to two negative signals corresponding to both signals of H_2_′′/H_6_′′ (7.20 and 7.55 ppm) and a positive one for both signals of N=CH and H_3_′′/H_5_′′, presenting a positive NOE of 9% and 8%, respectively (Figure 3). Curiously, only the H_2_′′/H_6_′′ signal shifted at 7.55 ppm inside the multiplet, and no other hydrogen presented a negative signal , showing that the inversion of phase was not an artifact of the technique due to the proximity of signals (Figure 3).

Additionally, we obtained a 2D-NOESY spectrum and verified that each expected diagonal signal from a conformer presented a cross-correlation in-phase with the correspondent signal of the other conformer (supplementary material). This effect was dependent on the experimental mixing time (D8 of 40, 80 and 300 ms), with longer times resulting in more intense cross-correlations.

**Table 2:** Theoretical relative NOE obtained in MSpin software for the *E* and *Z* geometric isomers of LASSBio-1083 (**1**). 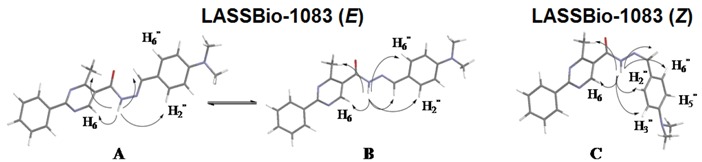

Form	N H–N=CH	N H–H_6_	N H–H_2_′′ ^a^	N H–H_3_′′	N H–H_6_′′ ^a^	N H–CH_3_
**A**	0.063	0.650	0.033	-	-	0.015
**B**	0.746	0.497	0.006	-	0.014	0.018
**C**	0.064	0.586	0.811	0.023	0.022	0.017

^a^ The pair H_2_” and H_6_” is chemically identical in a dynamic model, but the software only considers a static form and gives different values for pairs of equal hydrogens.

Normally this type of cross-correlation can be attributed to an artifact because of a chemical exchange with solvent. However, this is not the case, as we observed a cross-correlation for all protons including those that were not exchangeable. The cross-correlation is most plausibly due to an exchange among multiple conformers in which a ^1^H has a distinct chemical shift in each conformer. The time required for one conformer to become another explains the increase in intensity with longer mixing times.

These NOE experiment results led to the hypothesis that each signal could be related to the presence of two conformers. Moreover, we could exclude the *Z* stereoisomer because all NOE results are in agreement with the more stable *E* stereoisomer [20], as theoretically predicted. Thus, the hydrogen of NH could be *synperiplanar* (*sp*) or *antiperiplanar* (*ap*) in relation to the carbonyl oxygen atom of the *E* isomer (Figure 3). This hypothesis was corroborated by HPLC studies in which only one compound was detected as well as by the change in the ratio between the pair of duplicated signals when CDCl_3_ was used in place of DMSO-*d*_6_ for the ^1^H-NMR analysis, as previously observed by Palla and coworkers for alkyl-NAH [7].

#### 2.2.2. NAH Derivative 1 Conformers Determination

**Figure 4:**
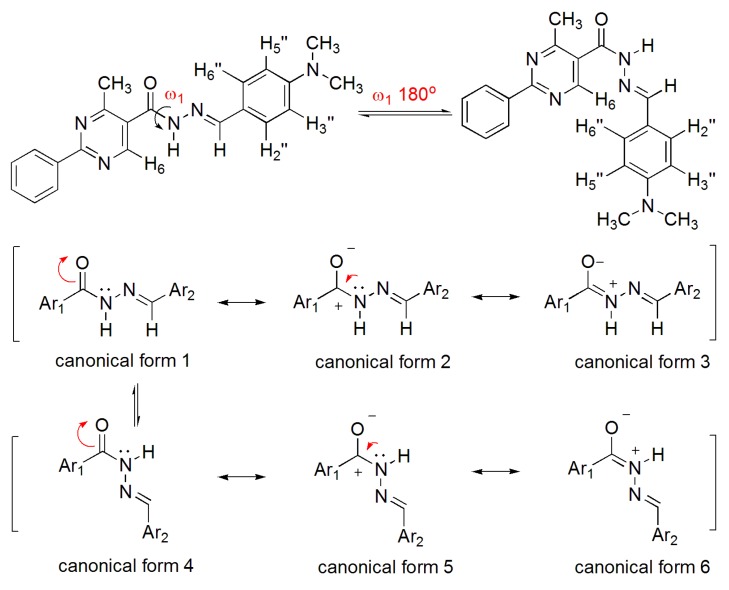
Representation of *ap*/*sp* conformers of the amide bond of NAH derivative **1** and its canonical forms.

**Figure 5:**
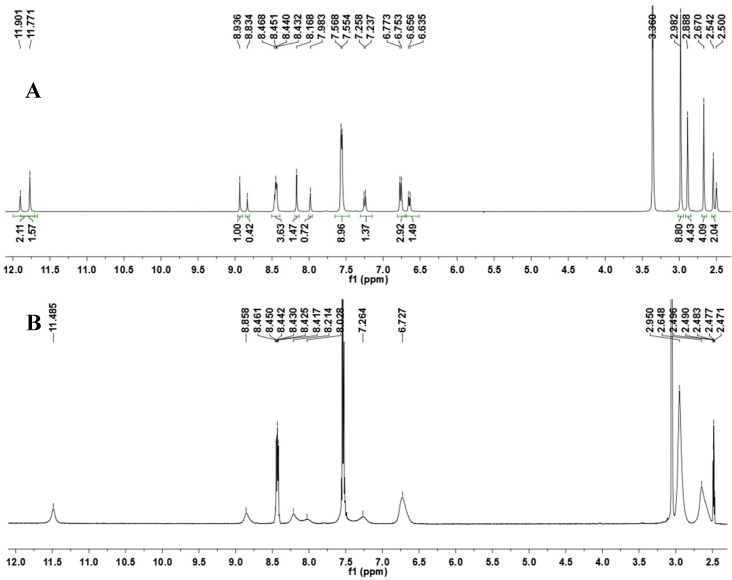
^1^H-NMR spectra of the NAH derivative **1** at 20 °C (**A**) and 80 °C (**B**).

Based on the work of Quattropani and colleagues [10], who described the presence of conformers for alkyl NAH compounds (Figure 4), we carried out a ^1^H-NMR experiment in DMSO-*d*_6_ at 80 °C to determine whether the coalescence of duplicated signals would be observed (Figure 5). A complete coalescence was observed for the NH, H_6_, H_3_′′/H_5_′′, N(CH_3_)_2_ and CH_3_ signals, while partial coalescence was observed for N=CH and H_2_′′/H_6_′′. The reversibility of these changes was verified when the experimental temperature was returned to 20 °C. This result corroborated the hypothesis of the conformers: the energy required to faster overcome the rotational barrier is reached upon an increase in temperature, leading to a rapid conversion between the conformers. Traditionally, the amide group is represented by the canonical forms **1** and **3**.

The resonance effect between the lone pair on the nitrogen sp^2^ atom and the carbonyl π bond contributes to the double bond character of the amides in the planar form. In this context, the resonance structure 3 is considered more significant for the double bond character and rotational barrier [21]. 

Converting a conformer into another requires rotation around the C-N bond, which consequently changes the nitrogen sp^2^ hybridization to a pyramidal arrangement. In the pyramidal geometry, the lone pair of nitrogen is placed in an orbital with high s character for stabilization. The disruption of favorable interactions between N and C raises the rotational barrier. In the case of formamide, the barrier is 18 kcal mol^−1^ [22,23,24,25].

Wiberg *et al*. conducted theoretical studies concerning the amide bond rotation. Their calculations showed that the C-N bond lengthens by 0.08 Ǻ upon rotation from the planar form to the transition state, whereas the C-O bond shortens slightly (by 0.01 Ǻ). Moreover, an insignificant difference in charge density at the oxygen was observed in the planar form and the transition state. Hence, the oxygen is assumed to simply polarize the C-O bond, resulting in a large δ^+^ on the carbon [22].

As the earlier model with two canonical structures did not explain these results, one that incorporated a carbonyl dipolar canonical form was suggested (2). Therefore, the rotational barrier is attributed mainly to the resonance structures **2**/**3** and **5**/**6** [22,23].

**Figure 6:**
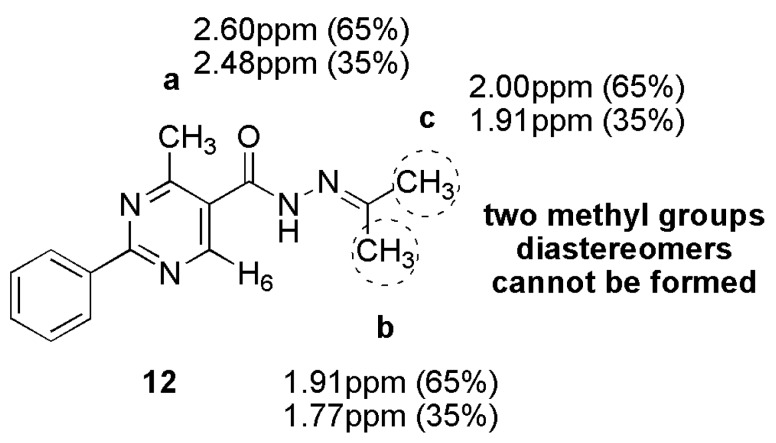
^1^H-NMR signal duplications and its proportions for the methyl groups in NAH derivative **12**.

*N*-Methylation of NAH **1**, which results in *N*-methyl-NAH **3a**, causes conformational restriction of the amide bond, thus preventing its rotation, as previously described by our group [17,26]. In fact, this modification abolished the duplication of the signals in the NMR spectra. Another approach used to evaluate the presence of two conformers was the synthesis of isopropylidene hydrazones. The latter was based on the works of Wyrzykiewicz and Prukala [27] and Palla [5], to eliminate the possibility of forming *E*/*Z* stereoisomers. The compound (isopropylidene)-2-phenyl-4-methyl-pyrimidine-5-hydrazide (**12**) (Figure 6) was synthesized by condensation of 2-phenyl-4-methyl-pyrimidine-5-hydrazide (**11a**) with acetone. Its ^1^H-NMR spectrum presented two singlets for each NH, H_6_ and CH_3_**a** and another three for CH_3_**b** and CH_3_**c**. Regarding CH_3_**a**, the signal present to a lesser extent showed the same chemical shift of the solvent DMSO-*d*_6_ (2.48 ppm). Likewise, the signal for the smaller proportion of CH_3_**c** and that for the greater proportion of CH_3_**b** also coincided (both were at 1.9 ppm). This result confirmed the presence of conformers.

Considering the proportion of the conformers, we believe that the more deshielded signal from N=CH matches the conformer *ap*, based on the work by Palla in which the more deshielded signal was attributed to imine hydrogen of conformer *ap* in DMSO-*d*_6_ and in CDCl_3_ [7]. Furthermore, in the *ap* conformation, the two dipolar moments (C^δ^^+^=O^δ−^ and N^δ−^-H^δ^^+^) are aligned, which does not occur in the *sp* conformer [21]. 

According to literature reports, the presence of conformers for the NAH compounds is typically observed in those with a spacer between the carbonyl group and the aromatic ring or in alkyl-*N*-acylhydrazones. However, NAH compound **1** does not structurally match the compounds described in previous reports. Perhaps an *ortho* steric effect, exerted by the methyl group on the carbonyl, and/or an electronic effect induced by the pyrimidine ring could be related to the observed phenomenon [28]. Thus, we decided to study the stereoelectronic requirements for the existence of conformers.

#### 2.2.3. Stereoelectronic Effects on NAH Derivative (**1**) *ap*-*sp* Amide Bond Rotamers

To evaluate the *ortho* steric effect, the methyl group was replaced by an H atom (**2a**) and an ethyl group (**2b**). The first modification represents the absence of any steric effect while the second one increases the steric effect. Both compounds showed duplicated signals in the ^1^H-NMR spectra, as shown in Table 3. Additionally, the pyrimidine ring was replaced with a phenyl ring to verify the influence of the electronic effect on the amide bond, generating compounds **2c** and **2d**. Thus, we could study the influence of the heterocyclic ring both in the presence (**2c**) and absence (**2d**) of an *ortho* steric effect. Only **2d** did not show duplicated signals. Therefore, we found that the steric effect and/or the pyrimidine ring induced the formation of conformers in this class of NAH.

**Table 3:** Chemical shifts (ppm) and relative integration (between parentheses) of the main hydrogen signals for the *N*-acylhydrazone derivatives **1** and **2a**–**d**. 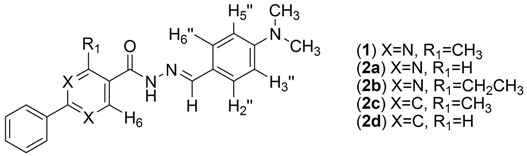

cpd ^a^	CO-N H		H_6_		N=C H		H_2_′′/H_6_′′		H_3_′′/H_5_′′		N(CH_3_)_2_		R_1_	
**1** **^b^**	11.79 (34)	11.67 (66)	8.91 (66)	8.81 (34)	8.17 (66)	7.98 (34)	7.55 (66)	7.24 (34)	6.76 (66)	6.64 (34)	2.97 (66)	2.88 (34)	2.66 (66)	2.53(34)
**2a** **^c^**	11.91 (20)	11.87 (80)	9.31 ^e^	9.29 ^e^	8.31 (80)	8.02 (20)	7.60–7.58 ^d^ (80) ^g^	7.4 (20)	6.78 ^e^	6.73 ^e^	2.99 (80)	2.95 (20)	-	-
**2b** **^b^**	11.86 (37)	11.74 (63)	8.90 (63)	8.79 (37)	8.15 (63)	7.9 (37)	7.56–7.53 ^d^ (63) ^g^	7.21 (37)	6.75 (63)	6.62 (37)	2.97 (63) ^g^	2.87 (37)	-	-
**2c** **^c^**	11.54 (17)	11.51 (83)	7.75–7.70 multiplet		8.17 (83)	7.95 (17)	7.61–7.39 ^d^ (83) ^g^	7.23 (17)	6.77 (83)	6.64 (17)	2.99 (83)	2.90 (17)	2.46 (83)	2.34 (17)
**2d** **^b^**	11.60		8.00 ^f^		8.34		7.54–7.38 ^d^		6.77		2.98		8.00 ^f^	

^a^ cpd = compound; ^b^ 40 °C; ^c^ 20 °C; ^d^ inside a multiplet; ^e^ signals are not totally separated; ^f^ represents 2H because R_1_ = H; ^g^ calculated by the difference from the other conformer.

**Figure 7:**
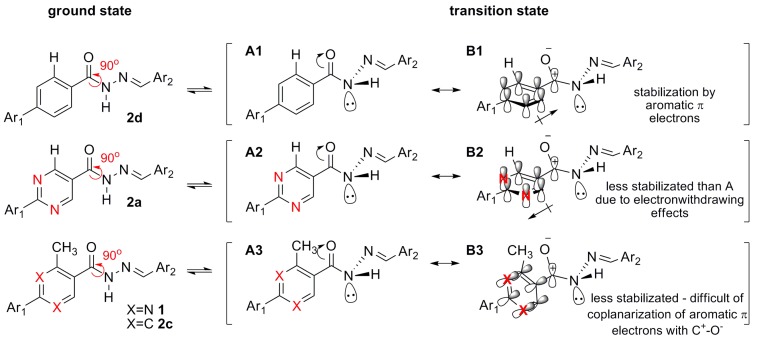
Resonance structure stabilization in the transition state of the amide bond rotamers.

Thus, the decoplanarization between the carbonyl and the aromatic ring (induced by the *ortho*-alkyl group) as well as the electron withdrawing effect exerted by the pyrimidine ring on the carbonyl group increase polarization. Both effects are disadvantageous in regard to the transition state between one conformer and the other. The transition state between amide rotamers is when the C-N bond is turned 90° and the nitrogen is not conjugated with the carbonyl (resulting in a pyramidalized N atom). As stated by Olsen and colleagues for nicotinamide and picolinamide, in the transition state, the π electrons from the aromatic ring can stabilize the resonance forms **B**, lowering the transition state energy and facilitating amide bond rotation (Figure 7). As shown, **2d** and **2a** are capable of coplanarizing their aromatic π electrons in the **B** canonical form with C^+^-O^−^. However, while this feature stabilizes the canonical form **B1** in compound **2d**, the recognized electron withdrawing effect of pyrimidine reduces stabilization (form **B2** in compound **2a**), leading to increases in the transition state energy and rotational barrier (Figure 7). This type of effect is supported by the observed 5.4 kcal/mol difference in the amide bond rotational barrier between nicotinamide and picolinamide [25]. Additionally, due to the more difficult coplanarization generated by *ortho*-alkyl groups, compounds **1** and **2c** present a more energetic transition state **B3** and thus a higher rotational barrier.

The ^13^C-NMR spectra support these assumptions, as the carbon from the carbonyl bond is deshielded by approximately 2 and 3 ppm after the introduction of an *ortho*-alkyl group or the replacement of the pyrimidine ring with a phenyl, respectively (Table 4).

**Table 4:** ^13^C-NMR chemical shifts (ppm) of the carbonyl groups of NAH derivatives **1** and **2a**–**d**.

cpd	Ring	*Ortho* group	δ C=O (ppm)
**1**	pyrimidine	Me	167.42
**2a**	pyrimidine	H	165.47
**2b**	pyrimidine	Et	169.75
**2c**	phenyl	Me	164.83
**2d**	phenyl	H	162.91

The higher sp^2^ amide nitrogen character attributed to compounds **1** and **2a**–**d** was evaluated using HMBC, which allowed us to distinguish between the aromatic carbons directly attached to a carbonyl that is *cis* and those attached to a carbonyl that is *trans* to the amide N-^1^H three bonds away. Due to the local bond constraints and planarity, two vicinal bond angles (0° and 180°) are present in the stable conformers. The HMBC correlations show that for compounds **1** and **2a**–**c**, the signals of the amide N-^1^H of each conformer are coupled differently to the aromatic ^13^C (^13^C-CO-N-^1^H). Only the minor conformer, which has a *trans* correlation between these atoms, shows a ^3^*J*_CH_ coupling (Table 5). These results agree with the literature showing a higher coupling for the *trans* coupling compared to *cis* [29,30]. On the other hand, compound **2d** did not show a ^3^*J*_CH_ coupling in the same experiments, possibly due to the minor sp^2^ amide nitrogen character and the longer length of the C-N bond, which results in a decrease in the rotational barrier compared to the other compounds (**1**, **2a**–**c**). Because of this small barrier, **2d** does not possess the fixed *trans* conformation necessary for ^3^*J*_CH_ coupling in this series. In addition, ^3^*J*_CH_ coupling of the amide N-^1^H and imine ^13^C=N was present in all compounds, indicating the similar chemical environments with no great differences in conformation for this moiety of NAH compounds.

**Table 5:** HMBC for NAH derivatives **1**, **2a**, **2c** and **2d**.

cpd	Long-range correlations for the amide N-H			
	^2^ *J*_CH_ (maj)	^2^ *J*_CH_ (min)	^3^ *J*_CH_ (maj)	^3^ *J*_CH_ (min)
**1**	C=O	C=O	C=N	C=N, C-5
**2a**	C=O	C=O	C=N	C=N, C-5
**2c**	C=O	C=O	C=N	C=N, C-4
**2d**	C=O	^a^	C=N	^a^

^a^ compound **2d** presents only one signal.

The Gibbs free energy of activation (∆*G*^≠^) related to the transition state among one conformer and another can be calculated using Equation 1. Through a dynamic NMR process, we could estimate the coalescence temperature (T_c_), which provides, in association with the maximum peak separation (Δ*v* in Hz) at low-temperature (*i.e.*, slow exchange between conformers), the energy of the rotational barrier (∆*G*^≠^) [31]:

∆*G^≠^* = 4.57.10^−3^*T* (9.972 + log *T_c_*/Δ*v*) (1)

The ∆*G*^≠^ values showed that the steric effect exerted by the methyl group on the ring decoplanarization and the electron withdrawing effect of pyrimidine have similar effects on the rotational barrier because compounds **2a** and **2c** have almost the same ∆*G*^≠^ (Table 6). 

**Table 6:** Results for restricted rotation about the CO-NH bond of NAH derivatives **1** and **2a**–**c**.

cpd	Signal	*T*_c_ (K)	v_c_ (Hz)	*G*^≠^ (kcal mol^−1^)
**1**	N H	353.15	52.0	17.4
**2a**	N H	313.15	16.52	16.1
**2b**	N H	356.15	64.3	17.4
**2c**	N H	313.15	12.12	16.3

A small additional increase in the barrier was observed when both effects are present, as observed for **1** and **2b**, while no change was found when replacing the methyl group with an ethyl group. 

## 3. Experimental

### 3.1. General Procedures

NMR spectra were recorded on a 200/50 MHz Bruker DPX-200, 250/62.5 MHz Bruker DPX-250, 400/100 MHz Bruker DPX-400, 500/125 MHz Bruker Avance-500, 400/100 MHz Varian 400-Mr, 300/75 MHz Varian Unity-300 spectrometer. The spectra were performed in DMSO-*d_6_* and CDCl_3_ with TMS as internal standard. Chemical shifts (δ) are given in ppm and coupling constants (*J*) in Hertz. Melting points were determined with a Quimis Q340.23 apparatus and are uncorrected. Elemental analyses were carried out on a Thermo Scientific Flash EA 1112 Series CHN-Analyzer. Thin layer chromatography was performed on Merck Kieselgel 60 HF 254 plates and detection took place using UV (254 and 365 nm). All reagents and solvents were purchased from commercial suppliers and used without further purification.

### 3.2. Ethyl 4-methyl-2-phenylpyrimidine-5-carboxylate *(**4a**)*

In a round bottomed flask under argon atmosphere were successively added anhydrous ethanol (20 mL), sodium metal (0.745 g, 32.4 mmol), benzamidine hydrochloride (5.07 g, 32.4 mmol) and (*E*/*Z*)-ethyl 2-((dimethylamino)methylene)-3-oxobutanoate (6.00 g, 32.4 mmol). The reaction mixture was stirred at room temperature for 2 h. The solvent was evaporated under vacuum and the resulting residue was filtered and washed with cold water. The title compound was obtained as a white crystalline solid in 80% yield. ^1^H-NMR (200 MHz, DMSO-*d_6_*) δ 9.17 (s, 1H, H_6het_); 8.46–8.44 (m, 2H, H_2__′__Ph_, H_6__′__Ph_); 7.58–7.55 (m, 3H, H_3__′__Ph_, H_4__′__Ph_, H_5__′__Ph_); 4.36 (q, 2H, *J* = 8.0 Hz, OCH_2_); 2.80 (s, 3H, CH_3het_); 1.35 (t, 3H, *J* = 8.0 Hz, CH_3Et_). ^ 13^C-NMR (50 MHz, DMSO-*d_6_*) δ 168.13 (C=O); 164.50 (C_2het_); 164.31 (C_4het_); 158.90 (C_6het_); 136.11 (C_1′Ph_); 131.76 (C_4′Ph_); 128.84 (C_3′Ph_, C_5′Ph_); 128.37 (C_2′Ph_, C_6′Ph_); 121.30 (C_5het_); 61.30 (OCH_2_); 24.28 (CH_3het_); 14.01 (CH_3Et_).

### 3.3. Methyl 4-Ethyl-2-Phenylpyrimidine-5-Carboxylate *(**4b**)*

To a solution of benzamidine hydrochloride (0.085 g, 0.540 mmol) and sodium hydroxide (0.022 g, 0.550 mmol) in methanol (2 mL), was added (*E*/*Z*)-methyl 2-((dimethylamino)methylene)-3-oxopentanoate (0.10 g, 0.540 mmol). The reaction mixture was stirred at room temperature for 1 h, and then concentrated under reduced pressure. The precipitate was filtered and washed with cold water, affording the title compound as a white amorphous solid in 73% yield. ^1^H-NMR (400 MHz, DMSO-*d_6_*) δ 9.12 (s, 1H, H_6het_); 9.48–9.46 (m, 2H, H_2__′__Ph_, H_6__′__Ph_); 7.46–7.41 (m, 3H, H_3__′__Ph_, H_4__′__Ph_, H_5__′__Ph_); 3.89 (s, 3H, CH_3het_); 3.18 (q, 2H, *J* = 7.5 Hz, OCH_2_); 1.32 (t, 3H, *J* = 7.5 Hz, CH_3Et_). ^13^C-NMR (100 MHz, DMSO-*d_6_*) δ 172.98 (C=O); 165.42 (C_4het_); 159.19 (C_6het_); 136.88 (C_1′Ph_); 132.50 (C_4′Ph_); 128.88 (C_3′Ph_, C_5′Ph_); 128.50 (C_2′Ph_, C_6′Ph_); 120.16 (C_5het_); 52.14 (OCH_3_); 29.84 (CH_2_); 12.36 (CH_3_).

### 3.4. Ethyl 2-phenylpyrimidine-5-carboxylate *(**4c**)*

In a round bottomed flask under argon atmosphere were successively added anhydrous ethanol (5 mL), sodium metal (0.426 g, 18.5 mmol), benzamidine hydrochloride (2.90 g, 18.5 mmol) and diethyl 2-(ethoxymethylene)malonate (4.0 g, 18.5 mmol). The reaction mixture was stirred at room temperature for 9 h, and then poured into ice. The precipitate was filtered, washed with coldwater and recrystallized in EtOH-MeOH-CHCl_3_ (1:1:1) (45 mL), giving the ethyl 4-hydroxy-2-phenylpyrimidine-5-carboxylate (**8**) as a white crystalline solid in 50% yield. The derivative **8** (1.2 g, 4.91 mmol) and POCl_3_ (9.8 g, 63.9 mmol) were refluxed at 100^o^C for 1 h. The excess of POCl_3_ was removed under vacuum, ice was added on the resulting solid into the reaction flask, followed by filtration and washing with cold water. The chloride compound **9**, obtained as a white amorphous solid in 98% yield, was successively dehalogenated with zinc powder (0.176 g, 2.69 mmol, 4 equiv) in anhydrous THF (2 mL). The reaction mixture was stirred at 60 °C for 1 h, and then 5 drops of acetic acid were added to the reaction vessel. After stirring at 60 °C for 23 h, the reaction mixture was cooled to room temperature, followed by addition of CH_2_Cl_2_ (3 mL), filtration and evaporation of solvent. Purification by silica gel column chromatography (*n*-hexane-EtOAc 0.6%) afforded the title compound as a white crystalline solid in 50% yield. Compound **8**: ^1^H-NMR (200 MHz, DMSO-*d_6_*/TMS) δ 12.22 (s, 1H); 8.64 (s, 1H); 8.16 (d, 2H, *J* = 6.0 Hz); 7.68–7.51 (m, 3H); 4.25 (q, 2H, *J* = 6.0 Hz); 1.28 (t, 3H, *J* = 6.0 Hz). ^13^C-NMR (62.5 MHz, DMSO-*d_6_*) δ 163.9; 161.3; 160.1; 159.0; 133.0; 131.9; 129.1; 128.8; 115.0; 60.8; 14.5. Compound **9**: ^1^H-NMR (200 MHz, CDCl_3_/TMS) δ 9.19 (s, 1H); 8.50 (dd, 2H, *J* = 2.2 Hz, 8.0 Hz); 7.61–7.46 (m, 3H); 4.46 (q, 2H, *J* = 7.0 Hz); 1.45 (t, 3H, *J* = 7.0 Hz). ^13^C-NMR (50 MHz, CDCl_3_/TMS) δ 166.57; 162.94; 160.61; 160.52; 135.32; 132.48; 129.29; 128.85; 121.61; 62.28; 14.23. Compound **4c**: ^1^H-NMR (250 MHz, DMSO-*d_6_*/TMS) δ 9.27 (s, 2H, H_4het_, H_6het_); 8.45–8.41 (m, 2H, H_2′Ph_, H_6′Ph_); 7.59–7.52 (m, 3H, H_3′Ph_, H_4′Ph_, H_5′Ph_); 4.36 (q, 2H, *J* = 7.5 Hz, OCH_2_); 1.33 (t, 3H, *J* = 7.5 Hz, CH_3_). ^13^C-NMR (63 MHz, DMSO-*d_6_*/TMS) δ 166.30 (C=O); 163.84 (C_2het_); 158.67 (C_4het_, C_6het_); 136.49 (C_1′Ph_); 132.45 (C_4′Ph_); 129.37 (C_3′Ph_, C_5′Ph_); 128.90 (C_2′Ph_, C_6′Ph_); 122.28 (C_5het_); 61.87 (OCH_2_); 14.47 (CH_3_).

### 3.5. Methyl 3-methyl-biphenyl-4-carboxylate *(**4d**)*

In a round bottomed flask into ice-bath, were added methanol (15 mL), acetyl chloride (8.72 g, 111 mmol) and 4-bromo-2-methylbenzoic acid (1.0 g, 4.65 mmol). The reaction mixture was stirred at room temperature for 24 h, and then concentrated under reduced pressure. The resulting residue was extracted with CH_2_Cl_2_. The organic layer was washed with 10% sodium carbonate solution, dried over anhydrous Na_2_SO_4_, filtered and evaporated, giving the methyl 4-bromo-2-methylbenzoate **10** as a colorless oil in 86%, which was directly used in cross coupling reaction. The compound **10** (0.30 g, 1.31 mmol) was transferred to a round bottomed flask with a mixture of toluene-methanol (9:1, 4.5–0.5 mL), followed by addition of phenylboronic acid (0.192 g, 1.57 mmol), PdCl_2_(PPh_3_)_2_ (0.037 g, 4 mol %) and potassium carbonate (0.543 g, 3.93 mmol). The reaction mixture was stirred at 60 °C for 3 h, and after cooling to room temperature, was filtered over Celite. The resulting oil was purified by silica gel column chromatography (*n*-hexane-EtOAc 0%–1%), affording the title compound as a white crystalline solid in 91% yield, after evaporation under vacuum. Compound **10**: ^1^H-NMR (200 MHz, DMSO-*d_6_*/TMS) δ 7.73 (d, 1H, *J* = 8.0 Hz); 7.58 (s, 1H); 7.51 (d, 1H, *J* = 8.0 Hz); 3.82 (s, 3H); 2.49 (s, 3H). ^13^C-NMR (50 MHz, DMSO-*d_6_*/TMS) δ 167.18; 142.31; 134.65; 132.51; 129.54; 129.16; 126.33; 52.58; 21.18. Compound **4d**: ^1^H-NMR (200 MHz, CDCl_3_/TMS) δ 7.92 (d, 1H, *J* = 8.0 Hz, H_5Ph_); 7.55–7.51 (m, 2H, H_2Ph_, H_6Ph_); 7.40–7.25 (m, 5H, H_2__′__Ph_-H_6__′__Ph_); 3.83 (s, 3H, OCH_3_); 2.60 (s, 3H, CH_3_). ^13^C-NMR (50 MHz, CDCl_3_/TMS) δ 167.97 (C=O); 144.75 (C_1Ph_); 140.91 (C_1__′__Ph_); 140.14 (C_3Ph_); 131.36 (C_5Ph_); 130.49 (C_2Ph_); 128.95 (C_3__′__Ph_, C_5__′__Ph_); 128.30 (C_4Ph_); 128.09 C_4__′__Ph_); 127.33 (C_2__′__Ph_, C_6__′__Ph_); 124.47 (C_6Ph_); 51.89 (OCH_3_); 22.08 (CH_3_).

### 3.6. 4-Methyl-2-phenylpyrimidine-5-carbohydrazide *(**11a**)*

To a solution of ethyl 4-methyl-2-phenylpyrimidine-5-carboxylate (**4a**, 0.710 g, 2.92 mmol) in ethanol (5 mL) was added hydrazine monohydrate 100% (2.93 g, 2.84 mL, 58.4 mmol). The reaction mixture was stirred at 55 °C for 4 h. After cooling the reaction to room temperature, the round bottomed flask was placed in an ice-bath and the solid, filtered and washed with cold water. The title compound was obtained as a white crystalline solid in 83% yield. ^1^H-NMR (200 MHz, DMSO-*d_6_*) δ 9.80 (s, 1H, NH); 8.76 (s, 1H, H_6het_); 8.44–8.39 (m, 2H, H_2__′__Ph_, H_6__′__Ph_); 7.56–7.52 (m, 3H, H_3__′__Ph_-H_5__′__Ph_); 4.62 (s, 2H, NH_2_); 2.63 (s, 3H, CH_3_). ^13^C-NMR (50 MHz, DMSO-*d_6_*) δ 165.43 (C=O); 164.80 (C_2het_); 163.02 (C_4het_); 155.56 (C_6het_); 136.54 (C_1__′__Ph_); 131.16 (C_4__′__Ph_); 128.72 (C_3__′__Ph_, C_5__′__Ph_); 127.92 (C_2__′__Ph_, C_6__′__Ph_); 126.32 (C_5het_); 22.63 (CH_3_).

### 3.7. 4-Ethyl-2-phenylpyrimidine-5-carbohydrazide *(**11b**)*

To a solution of **4b** (0.191 g, 0.788 mmol) in ethanol (3 mL) was added hydrazine monohydrate 100% (0.789 g, 15.8 mmol). The reaction mixture was stirred at 50 °C for 5 h, cooled to room temperature and poured into ice. The solid was filtered and washed with cold water, giving the title compound as a white amorphous solid in 79% yield. ^1^H-NMR (400 MHz, DMSO-*d_6_*) δ 9.77 (s, 1H, NH); 8.72 (s, 1H, H_6het_); 8.42–8.40 (m, 2H, H_2__′Ph_, H_6__′Ph_); 7.54–7.51 (m, 3H, H_3__′Ph_, H_5__′Ph_); 4.59 (s, 2H, NH_2_); 2.90 (q, 2H, *J* = 8.0 Hz, CH_2_); 1.27 (t, 3H, *J* = 8.0 Hz, CH_3_). ^13^C-NMR (125 MHz, DMSO-*d_6_*) δ 170.12 (C=O); 165.40 (C_4het_); 163.66 (C_2het_); 156.09 (C_6het_); 137.23 (C_1_**_′_**_Ph_); 131.71 (C_4_**_′_**_Ph_); 129.31 (C_3_**_′_**_Ph_, C_5_**_′_**_Ph_); 128.44 (C_2__′Ph_, C_6__′Ph_); 126.68 (C_5het_); 28.62 (CH_2_); 13.14 (CH_3_). 

### 3.8. 2-Phenylpyrimidine-5-carbohydrazide *(**11c**)*

To a solution of ethyl 2-phenylpyrimidine-5-carboxylate (4c) (0.080 g, 0.350 mmol) in ethanol (2 mL) was added hydrazine monohydrate 100% (0.351 g, 7.01 mmol). The reaction mixture was stirred at room temperature for 2 h. Ice was added to the reaction flask and the solid, filtered and washed with cold water. The title compound was obtained as a white amorphous solid in 85% yield. ^1^H-NMR (250 MHz, DMSO-*d_6_*/TMS) δ 10.12 (s, 1H, NH); 9.20 (s, 2H, H_4het_, H_6het_); 8.43–8.39 (m, 2H, H_2′Ph_, H_6′Ph_); 7.55–7.52 (m, 3H, H_3′Ph_-H_5′Ph_); 4.64 (s, 2H, NH_2_). ^13^C-NMR (63 MHz, DMSO-*d_6_*/TMS) δ 165.04 (C=O); 162.88 (C_2het_); 156.71 (C_4het_, C_6het_); 136.78 (C_1′Ph_); 131.99 (C_4′Ph_); 129.31 (C_3′Ph_, C_5′Ph_); 128.56 (C_2′Ph_, C_6′Ph_); 125.03 (C_5het_). 

### 3.9. 3-Methyl-biphenyl-4-carbohydrazide *(**11d**)*

To a solution of methyl ester (4d) (0.60 g, 2.65 mmol) in ethanol (5 mL) was added hydrazine monohydrate 100% (3.98 g, 79.5 mmol). The reaction mixture was stirred at 50 °C for 15 h, and then concentrated under reduced pressure. Ice was added to the reaction flask and the solid, filtered and washed with cold water. The title compound was obtained as a white amorphous solid in 95% yield. ^1^H-NMR (200 MHz, DMSO-*d_6_*/TMS) δ 9.46 (s, 1H, NH); 7.69–7.66 (m, 2H, H_2Ph_, H_5Ph_); 7.54–7.37 (m, 6H, H_6Ph_, H_2′Ph_-H_6′Ph_); 4.49 (s, 2H, NH_2_); 2.42 (s, 3H, CH_3_). ^13^C-NMR (50 MHz, DMSO-*d_6_*/TMS) δ 168.85 (C=O); 141.68 (C_1Ph_); 140.04 (C_1′Ph_); 136.98 (C_3Ph_); 135.06 (C_4Ph_); 129.50 (C_3′Ph_, C_5′Ph_); 129.28 (C_5Ph_); 128.54 (C_2Ph_); 128.28 (C_4′Ph_); 127.26 (C_2′Ph_, C_6′Ph_); 124.25 (C_6Ph_); 20.08 (CH_3_).

### 3.10. Biphenyl-4-carbohydrazide *(**11e**)*

To a solution of methyl biphenyl-4-carboxylate (**4e**, 0.50 g, 2.36 mmol) in ethanol (5 mL) was added hydrazine monohydrate 100% (2.36 g, 47.1 mmol). The reaction mixture was stirred at 80 °C for 8 h, and then concentrated under reduced pressure. Ice was added to the reaction flask and the solid, filtered and washed with cold water. The title compound was obtained as a pale amorphous solid in 85% yield. ^1^H-NMR (200 MHz, DMSO-*d_6_*/TMS) δ 9.85 (s, 1H, NH); 7.93 (d, 2H, *J* = 8.0 Hz, H_3Ph_, H_5Ph_); 7.77–7.69 (m, 4H, H_2Ph_, H_6Ph_, H_2′Ph_, H_6′Ph_); 7.52–7.36 (m, 3H, H_3′Ph_-H_5′Ph_); 4.55 (s, 2H, NH_2_). ^13^C-NMR (50 MHz, DMSO-*d_6_*/TMS) δ 166.13 (C=O); 143.17 (C_1Ph_); 139.74 (C_1′Ph_); 132.66 (C_4Ph_); 129.57 (C_3Ph_, C_5Ph_); 128.55 (C_4′Ph_); 128.17 (C_3′Ph_, C_5′Ph_); 127.37 (C_2Ph_, C_6Ph_); 127.08 (C_2′Ph_, C_6′Ph_).

### 3.11. General Procedure to Synthesize the N-Acylhydrazones ***1, 2a**–**d***

To a solution of an aromatic cabohydrazide (1.10 mmol) in ethanol (5 mL) were added HCl catalytic (2 drops) and the appropriate aromatic aldehyde. The reaction mixture was stirring at room temperature for *ca* 30 min, and then poured into ice. The precipitate was filtered, washed with cold water, petroleum ether and recrystallized in EtOH. 

*N′-(4-(Dimethylamino)benzylidene)-4-methyl-2-phenylpyrimidine-5-carbohydrazide* (**1**): The condensation of 4-methyl-2-phenylpyrimidine-5-carbohydrazide (**11a**) and 4-dimethylamino- benzaldehyde, afforded the title compound as a yellow solid (mp. 206 °C) in 99% yield. ^1^H-NMR (200 MHz, DMSO-d_6_) δ 11.79, 11.67 (2s, 1H, NH); 8.91, 8.81 (2s, 1H, H_6het_); 8.45–8.41 (m, 2H, H_2′Ph_, H_6′Ph_); 8.17, 7.98 (2s, 1H, N=CH); 7.56–7.54 (m, 5H, H_3′Ph_–H_5′Ph_, H_2′′ap_, H_6′′ap_); 7.24 (d, 2H, H_2′′sp_, H_6′′sp_, *J* = 8.7 Hz); 6.76, 6.64 (2d, 2H, H_3′′_, H_5′′_, J = 8.7 Hz, 9.0 Hz); 2.97, 2.88 (2s, 6H, N(CH_3_)_2_); 2.66, 2.53 (2s, 3H, CH_3_). ^13^C-NMR (50 MHz, DMSO-d_6_) δ 167.42, 165.96 (C=O); 164.37, 163.48 (C_2het_); 162.95, 161.59 (C_4het_); 156.37, 156.10 (C_6het_); 151.97, 151.73 (C_4′′_); 149.60, 145.96 (N=CH); 136.97, 136.84 (C_1′Ph_); 131.47, 131.34 (C_4′Ph_); 128.99 (C_3′Ph_, C_5′Ph_); 128.26 (C_2′Ph_, C_6′Ph_, C_2′′_, C_6′′_); 127.64, 126.72 (C_5het_); 121.09, 121.03 (C_1′′_); 111.73 (C_3′′_, C_5′′_); 39.87 (N(CH_3_)_2_); 23.02, 22.89 (CH_3_). Anal. Calcd for C_21_H_21_N_5_O: C: 70.18; H: 5.89; N: 19.48. Found: C: 70.17; H: 5.89; N: 19.62.

*N′-(4-(Dimethylamino)benzylidene)-2-phenylpyrimidine-5-carbohydrazide* (**2a**): The condensation of 2-phenylpyrimidine-5-carbohydrazide (**11c**) and 4-dimethylaminobenzaldehyde, afforded the title compound as a yellow amorphous solid (mp. 269 °C) in 99% yield. ^1^H-NMR (300 MHz, DMSO-*d_6_*/TMS) δ 11.79 (broad s, 1H, NH); 9.28 (s, 2H, H_4het_, H_6het_); 8.47–8.44 (m, 2H, H_2′Ph_, H_6′Ph_); 8.30, 8.01 (2s, 1H, N=CH); 7.57–7.55 (m, 5H, H_3′Ph_, H_4′Ph_, H_5′Ph_, H_2′′_, H_6′′_); 7.39 (d, 2H, H_2′′_, H_6′′_, *J* = 4.0 Hz); 6.77–6.69 (m, 2H, H_3′′_, H_5′′_); 2.97, 2.92 (2s, 6H, N(CH_3_)_2_). ^13^C-NMR (75 MHz, DMSO-*d_6_*/TMS) δ 165.47, 165.33 (C=O); 164.66, 160.02 (C_2het_); 158.90, 157.43 (C_4_, C_6_); 152.45, 152.22 (C_4′′_); 150.42, 146.48 (N=CH); 137.11 (C_1′Ph_); 132.26 (C_4′Ph_); 129.54 (C_3′Ph_, C_5′Ph_); 129.35 (C_2′′_, C_6′′_); 128.90 (C_2′Ph_, C_6′Ph_); 126.70, 125.96 (C_5het_); 121.88 (C_1′′_); 112.50 (C_3′′_, C_5′′_); 40.56 (N(CH_3_)_2_). Anal. Calcd for C_20_H_19_N_5_O: C: 69.55; H: 5.54; N: 20.28. Found: C: 69.20; H: 5.54; N: 20.02.

*N′-(4-(Dimethylamino)benzylidene)-4-ethyl-2-phenylpyrimidine-5-carbohydrazide* (**2b**): The condensation of 4-ethyl-2-phenylpyrimidine-5-carbohydrazide (**11b**) and 4-dimethylamino- benzaldehyde, afforded the title compound as a yellow solid (mp. 240 °C) in 85% yield. ^1^H-NMR (200 MHz, DMSO-*d_6_*) δ 11.86, 11.74 (2s, 1H, NH); 8.90, 8.79 (2s, 1H, H_6het_); 8.47–8.43 (m, 2H, H_2′Ph_, H_6’Ph_); 8.15, 7.96 (2s, 1H, N=CH); 7.56–7.53 (m, 5H, H_3′Ph_–H_5′Ph_, H_2′′_, H_6′′_); 7.21 (d, 2H, H_2′′_, H_6′′_, *J* = 6.6 Hz); 6.75 (d, 2H, H_3′′_, H_5′′_, *J* = 6.6 Hz); 6.62 (d, 2H, H_3′′_, H_5′′_, *J* = 6.6 Hz); 2.99–2.94 (m, 8H, CH_2_, N(CH_3_)_2_); 2.87 (s, 6H, N(CH_3_)_2_); 2.81 (q, 2H, CH_2_, *J* = 7.5 Hz); 1.32–1.25 (m, 3H, CH_3_). ^ 13^C-NMR (50 MHz, DMSO-*d_6_*) δ 169.75, 167.93 (C=O); 167.22, 163.24 (C_4het_); 162.65, 161.25 (C_2het_); 155.93, 155.80 (C_6het_); 151.61, 151.36 (C_4′′_); 149.18, 145.50 (N=CH); 136.81, 136.67 (C_1′Ph_); 131.15, 131.00 (C_4′Ph_); 128.70, 128.56 (C_3′Ph_, C_5′Ph_); 127.92, 127.08 (C_2′′_, C_6′′_); 126.19 (C_2′Ph_, C_6′Ph_); 121.03 (C_1′′_), 120.95 (C_5het_); 111.69 (C_3′′_, C_5′′_); 39.64, 39.46 (N(CH_3_)_2_); 28.30, 28.08 (CH_2_); 12.49, 12.03 (CH_3_). Anal. Calcd for C_22_H_23_N_5_O: C: 70.76; H: 6.21; N: 18.75. Found: C: 70.62; H: 6.19; N: 18.80. 

*(E)-N′-(4-(Dimethylamino)benzylidene)-3-methylbiphenyl-4-carbohydrazide* (**2c**): The condensation of 3-methyl-biphenyl-4-carbohydrazide (**11d**) and 4-dimethylaminobenzaldehyde, afforded the title compound as a yellow amorphous solid (mp. 238 °C) in 94% yield. ^1^H-NMR (300 MHz, DMSO-*d_6_*/TMS) δ 11.41 (s, 1H, NH); 8.17, 7.94 (2s, 1H, N=CH); 7.72–7.67 (m, 2H, H_2Ph_, H_5Ph_); 7.58–7.35 (m, 8H, H_6Ph_, H_2′Ph_-H_6′Ph_, H_2′′_, H_6′′_); 7.22 (d, 2H, H_2′′_, H_6′′_, *J* = 8.4 Hz); 6.74, 6.62 (2d, 2H, H_3′′_, H_5′′_, *J* = 8.9 Hz, *J* = 8.4 Hz); 2.96, 2.87 (2s, 6H, N(CH_3_)_2_); 2.45, 2.33 (2s, 3H, CH_3_). ^13^C-NMR (75 MHz, DMSO-*d_6_*/TMS) δ 164.83 (C=O); 151.74, 151.48 (C_4′′_); 148.44, 144.70 (N=CH); 141.66, 140.62 (C_1Ph_); 139.69 (C_1′_); 136.84, 135.66 (C_3Ph_); 134.82 (C_4Ph_); 129.17, 129.06 (C_3′Ph_, C_5′Ph_, C_2′′_, C_6′′_); 128.63, 128.32 (C_2′Ph_, C_6′Ph_); 127.85 (C_4′Ph_); 126.97 (C_2Ph_, C_5Ph_); 124.03, 123.46 (C_6Ph_); 121.84 (C_1′′_); 112.03, 111.29 (C_3′′_, C_5′′_); 39.95 (N(CH_3_)_2_); 19.69 (CH_3_). Anal. Calcd for C_23_H_23_N_3_O: C: 77.28; H: 6.49; N: 11.76. Found: C: 76.92; H: 6.47; N: 11.59.

*(E)-N′-(4-(Dimethylamino)benzylidene)-biphenyl-4-carbohydrazide* (**2d**): The condensation of biphenyl-4-carbohydrazide (**11e**) and 4-dimethylaminobenzaldehyde, following the general procedure for the synthesis of *N*-acylhydrazones, afforded the title compound as a yellow amorphous solid (mp. 289 °C) in 99% yield. ^1^H-NMR (300 MHz, DMSO-*d_6_*/TMS) δ 11.58 (s, 1H, NH); 8.32 (s, 1H, N=CH); 7.98 (d, 2H, H_3Ph_, H_5Ph_, *J* = 8.0 Hz); 7.8 (d, 2H, H_2Ph_, H_6Ph_, *J* = 8.0 Hz); 7.74–7.72 (m, 2H, H_2′Ph_, H_6′Ph_); 7.55–7.47 (m, 4H, H_2′′_, H_6′′_, H_3′Ph_, H_5′Ph_); 7.42–7.38 (m, 1H, H_4′Ph_); 6.75 (d, 2H, H_3′′_, H_5′′_, *J* = 8.0 Hz); 2.96 (s, 6H, N(CH_3_)_2_). ^13^C-NMR (75 MHz, DMSO-*d_6_*/TMS) δ 162.27 (C=O); 151.52 (C_4′′_); 148.64 (N=CH); 142.97 (C_1Ph_); 139.12 (C_1′Ph_); 132.53 (C_4Ph_); 129.02 (C_3Ph_, C_5Ph_); 128.42 (C_3′Ph_, C_5′Ph_); 128.14 (C_2′′_, C_6′′_); 126.85 (C_2Ph_, C_6Ph_, C_4′Ph_); 126.58 (C_2′Ph_, C_6′Ph_); 121.62 (C_1′′_); 111.81 (C_3′′_, C_5′′_). Anal. Calcd for C_22_H_21_N_3_O: C: 76.94; H: 6.16; N: 12.24. Found: C: 76.65; H: 6.15; N: 11.96. 

### 3.12. (E)-N′-(4-(Dimethylamino)Benzylidene)-N,4-Dimethyl-2-Phenylpyrimidine-5-Carbohydrazide *(**3a**)*

To a solution of *N*-acylhydrazone 1 (0.14 mmol) and potassium carbonate (0.42 mmol) in acetone (5 mL) in an ice-bath was added methyl iodide (0.42 mmol). After, the reaction mixture was stirred at 43 °C for 24 h. The addition of 0.42 mmol of methyl iodide was needed, and then the reaction mixture was stirred at the same temperature for more 20 h. The reaction mixture was concentrated under vacuum, ethanol (1 mL) was added and the resulting mixture poured into ice. After filtering, the solid was heated into petroleum ether, and then refiltered to give the title compound as a yellow amorphous solid (mp. 188–189 °C) in 77% yield. ^1^H-NMR (300 MHz, DMSO-*d_6_*/TMS) δ 8.73 (s, 1H, H_6het_); 8.46–8.42 (m, 2H, H_2′Ph_, H_6′Ph_); 7.94 (s, 1H, N=CH); 7.54–7.52 (m, 3H, H_3′Ph_-H_5′Ph_); 7.22 (d, 2H, *J* = 9.0 Hz, H_2′′_, H_6′′_); 6.61 (d, 2H, *J* = 9.0 Hz, H_3′′_, H_5′′_); 3.50 (s, 3H, N-CH_3_); 2.85 (s, 6H, N(CH_3_)_2_); 2.43 (s, 3H, CH_3_). ^13^C-NMR (75 MHz, DMSO-*d_6_*/TMS) δ 167.81 (C=O); 164.18 (C_2het_); 163.12 (C_4het_); 156.09 (C_6het_); 152.13 (C_4′′_); 143.46 (N=CH); 137.53 (C_1′Ph_); 131.65 (C_4′Ph_); 129.57 (C_3′Ph_, C_5′Ph_); 129.36 (C_2′′_, C_6′′_); 128.76 (C_2′Ph_, C_6′Ph_); 128.62 (C_5het_); 122.34 (C_1′′_); 112.50 (C_3′′_, C_5′′_); 40.32 (N(CH_3_)_2_; 28.92 (N-CH_3_); 23.29 (CH_3_). Anal. Calcd for C_22_H_23_N_5_O: C: 70.76; H: 6.21; N: 18.75. Found: C: 70.41; H: 6.05; N: 18.43. 

### 3.13. 4-Methyl-2-Phenyl-N’-(Propan-2-Ylidene)Pyrimidine-5-Carbohydrazide *(**12**)*

A mixture of 4-methyl-2-phenylpyrimidine-5-carbohydrazide (11a) 0.07 g, 0.31 mmol) in acetone (7 mL) was stirred for 15 h at 45 °C. After, the solvent was evaporated to give a white solid in 85% yield (mp. 186–188 °C). ^1^H-NMR (400 MHz, DMSO-*d_6_*/TMS) δ 10.9, 10.6 (2s, 1H, NH); 8.83, 8.70 (2s, 1H, H_6het_); 8.42–8.40 (m, 2H, H_2′Ph_, H_6′Ph_); 7.54–7.53 (m, 3H, H_3′Ph_-H_5′Ph_); 2.60, 2.48 (2s, 3H, CH_3_); 1.99, 1.91, 1.77 (3s, 6H, N=C-(CH_3_)_2_). ^13^C-NMR (125 MHz, DMSO-*d_6_*/TMS) δ 168.57, 165.73 (C=O); 164.32, 163.47 (N=C); 162.97, 162.39 (C_2het_); 159.30, 153.61 (C_4het_); 156.47, 156.30 (C_6het_); 137.15, 137.12 (C_1′Ph_); 131.72, 131.60 (C_4′Ph_); 129.31, 129.25 (C_3′Ph_, C_5′Ph_); 128.43, 128.38 (C_2′Ph_, C_6′Ph_); 127.43 (C_5het_); 25.62, 25.56 (N=C-CH_3_); 23.14, 23.09 (CH_3het_); 18.55, 17.98 (N=C-CH_3_). Anal. Calcd for C_15_H_16_N_4_O: C: 67.15; H: 6.01; N: 20.88. Found: C: 66.93; H: 6.05; N: 20.52.

### 3.14. Molecular Modeling

The sketching, geometry optimization and conformational search of compounds were performed in PC Spartan Pro 1.0.5 software. They were subjected to structural minimization by the use of molecular mechanics, using the base MMFF. Subsequently, the conformational analysis was performed, using the semi-empirical AM1.

The NOE prediction study was performed by selecting the minimum conformation of each stereoisomer (*E*/*Z*), and additionally the election of a second conformer (B) for the *E* isomer which ΔHf = 114.68 kcal/mol. Conformers that have the carbonyl group *synperiplanar* related to NH hydrogen were obtained by a dihedral angle search in 12 steps of 30° on the amide bond CO-NH, leaving the other angles free. Then we selected the lowest energy conformer, generated by the conformational analysis with the semi-empirical AM1 method, which presented the carbonyl *synperiplanar* related to the NH. The NOE calculations were carried out in the MSpin 1.03 software from the selected structures from the conformational analysis.

## 4. Conclusions

In this work, we revealed that duplicated signals observed in NMR spectra of 4-methyl-2-phenylpyrimidine-N-acylhydrazone correspond to the presence of two CO-NH bond- related *syn* and *antiperiplanar* conformers. To our knowledge, this was the first description of conformers in aryl-NAH compounds. Our assumptions were based on NMR data from NOEdiff spectra of NH and phenyl H_2’’_/H_6’’_ irradiations, 2D-NOESY and dynamic ^1^H NMR and also by synthesis of isopropylidene hydrazone. The possibility of conformer’s observation rises from the increase of rotational barrier (∆*G*^≠^) among them, which results from both the decoplanarization of the aromatic ring and carbonyl group (induced by an *ortho-*alkyl group) and the electron withdrawing nature of the pyrimidine ring.

## Supplementary Materials

Supplementary materials can be accessed at: http://www.mdpi.com/1420-3049/18/10/11683/s1.
